# Platelet-Rich Plasma as an Alternative to Xenogeneic Sera in Cell-Based Therapies: A Need for Standardization

**DOI:** 10.3390/ijms23126552

**Published:** 2022-06-11

**Authors:** Eduardo Anitua, Mar Zalduendo, Maria Troya, Mohammad H. Alkhraisat, Leticia Alejandra Blanco-Antona

**Affiliations:** 1 BTI—Biotechnology Institute, 01007 Vitoria-Gasteiz, Spain; marimar.zalduendo@bti-implant.es (M.Z.); maria.troya@bti-implant.es (M.T.); mohammad.hamdan@bti-implant.es (M.H.A.); 2 University Institute for Regenerative Medicine and Oral Implantology—UIRMI (UPV/EHU-Fundación Eduardo Anitua), 01007 Vitoria-Gasteiz, Spain; 3 PhD Program, Department of Surgery, Faculty of Medicine, University of Salamanca, C/Alfonso X El Sabio s/n, 37007 Salamanca, Spain; 4 Department of Surgery, Faculty of Medicine University of Salamanca, C/Alfonso X El Sabio s/n, 37007 Salamanca, Spain; lblanco@usal.es

**Keywords:** platelet-rich plasma, cell therapy, PRGF, stem cells, xenogeneic supplements, regenerative medicine, cell culture

## Abstract

There has been an explosion in scientific interest in using human-platelet-rich plasma (PRP) as a substitute of xenogeneic sera in cell-based therapies. However, there is a need to create standardization in this field. This systematic review is based on literature searches in PubMed and Web of Science databases until June 2021. Forty-one studies completed the selection criteria. The composition of PRP was completely reported in less than 30% of the studies. PRP has been used as PRP-derived supernatant or non-activated PRP. Two ranges could be identified for platelet concentration, the first between 0.14 × 10^6^ and 0.80 × 10^6^ platelets/µL and the second between 1.086 × 10^6^ and 10 × 10^6^ platelets/µL. Several studies have pooled PRP with a pool size varying from four to nine donors. The optimal dose for the PRP or PRP supernatant is 10%. PRP or PRP-derived supernatants a have positive effect on MSC colony number and size, cell proliferation, cell differentiation and genetic stability. The use of leukocyte-depleted PRP has been demonstrated to be a feasible alternative to xenogeneic sera. However, there is a need to improve the description of the PRP preparation methodology as well as its composition. Several items are identified and reported to create guidelines for future research.

## 1. Introduction

Cell therapy represents a promising alternative approach to repair damaged tissues in many clinical applications where the use of biomaterials may not be sufficient [1]. Mesenchymal stem cells (MSCs) are frequently, but not always, the primary cell source in regenerative medicine. They are good candidates due to their great ability of self-renewal and multilineage differentiation along with strong immunosuppressive properties [2,3]. Human MSCs can be isolated from several tissues, mainly bone marrow, adipose tissue and umbilical cord blood. However, their low prevalence makes them insufficient for clinical applications without prior ex vivo expansion [4].

Currently, fetal bovine serum (FBS), also referred to as fetal calf serum (FCS), is the most widely used cell culture supplement in both the research and clinical fields [5]. However, the use of xenogeneic products involves many safety and regulatory concerns. FBS is an ill-defined supplement with great variability among batches. Its use entails a risk of zoonotic transmission of contaminants, such as viruses or prions, and possible adverse immunological reactions due to xenogeneic components. Additionally, obtaining blood from the animals involves certain ethical and welfare issues [4,6]. All these concerns demand suitable alternatives to develop new culture supplements for clinical application following the Good Manufacturing Practice (GMP). In this sense, from 1979 until the present date, an increasing amount of experimental works have been caried out using FBS substitutes as culture medium supplements, with the majority of the articles having been published by researchers in USA, China and Italy [7]. On the other hand, annually, more than 2 million bovine fetuses are used worldwide to produce approximately 800,000 L of FBS for biological research, clinical trials, and pharmaceutical production. However, there is a simultaneous increasing demand with a restricted supply due to climate change and the reduction in livestock reserves. Consequently, FBS costs have been significantly increased (over 300% in recent years as of 2016) as well as questionable practices in production having been adopted [5]. In this unfavorable context, PRP becomes a promising alternative to FBS for cell expansion, reducing the risk of xenoimmunization and zoonotic transmission as well as the cost of its acquisition [8].

Platelet-rich plasma provides an interesting tool to influence the cells and trigger changes that activate several physiological processes that conclude in tissue healing [9,10]. Indeed, platelets contribute to this by the release of physiologically balanced biomolecules that can orchestrate cell behavior in terms of growth, recruitment, differentiation and morphogenesis. These biomolecules are sourced from platelet granules (alpha, delta and lambda granules) and plasma [11]. Platelets interact with cells by the release of growth factors upon binding to their receptors on the cell surface. For example, platelet-derived growth factor (PDGF) interacts with mesenchymal cells (such as fibroblasts, osteoblasts and adipocytes) that express α- and β-type receptors [12]. These receptors participate in the transduction of proliferative stimulus and β-type receptors participate in the transduction of chemotaxis [13]. Another important mediator of cell communication is beta-transforming growth factor (β-TGF), which participates in all physiological processes [14]. Most of the cells express receptors for this growth factor that induces mesenchymal stem cells to proliferate and differentiate [15]. It is an angiogenic factor. However, it has an inhibitory effect on osteoclast formation and epithelial cell proliferation [16]. The epidermal growth factor (EGF) induces epithelial cell and fibroblast recruitment and proliferation. It plays an important role in the synthesis of the granulation tissue. For example, a high number of EGF receptors are expressed by pre-chondrocytes, fibroblasts and pre-osteoblasts [17]. In this regard, fibronectin interacts with cells as well as components of the extracellular matrix to promote cell proliferation and migration in order to replace the blood clot by the provisional matrix [18]. Basic-fibroblastic growth factor (b-FGF or FGF-2) is a mitogenic and angiogenic factor that orchestrates the proliferation of mesenchymal stem cells [19,20]. Insulin-like growth factor-I (IGF-I) is pro-angiogenic and induces the proliferation of pre-osteoblasts and the extracellular matrix formation by osteoblasts [21,22,23]. It influences mesenchymal stem cell proliferation and differentiation during the generation of cartilage, adipose tissue, muscles and neurons [24,25]. Angiopoietin-2 and vascular endothelial growth factor (VEGF) work together to promote angiogenesis [26]. Interestingly, platelets release, for example, platelet factor 4 (PF4) that inhibits angiogenesis probably to control angiogenesis [16]. VEGF is a mitogenic factor and stimulates the differentiation of different cells (such as fibroblasts, epithelial cells and renal cells) [27]. Platelet-released nucleotides (ATP and ADP) activate other platelets and participate in all phases of tissue healing. The latter is supported by the expression of P2 receptors (binds extracellular nucleotides) in almost all cell types [28]. Platelets store 95% of the neurotransmitter serotonin present in the blood. It is a mitogenic factor (for example, hepatocyte- and osteoblast-like cells) that participates in tissue remodeling [29,30,31,32]. Moreover, platelets release biomolecules (thrombocidins, PF4, RANTES, platelet basic protein and thymosin beta 4) that mediate their anti-microbial potential [33,34]. Other molecules, such as interleukin 4 (IL-4), hepatocyte growth factor and tumour necrosis factor alpha (TNF-α), could regulate inflammation through the inhibition of the activation of transcription factor kappa-B (NF-κB) and the expression of cyclooxygenase 2 (COX2) and C-X-C chemokine receptor type 4 (CXCR4) [35].

During the processes of activation and apoptosis, platelets release extracellular vesicles (EVs). The International Society for Extracellular Vesicles (ISEV) defines exosomes as the smallest extracellular vesicles, bound by a lipid bilayer and without a functional core, released through an endocytic process [36]. EVs constitute a heterogeneous population of membrane vesicles consisting of exosomes (30–150 nm), microvesicles (100–1000 nm) and apoptotic bodies (100–5000 nm). These vesicles carry important bioactive molecules, including proteins, lipids and mitochondrial DNA, as well as miRNA, long non-coding RNA and mRNA. These vesicles can be taken up by other cells, which introduces another level of complexity in terms of intercellular signaling [37]. Each type of EV has unique characteristics, and its composition represents the cell type of origin and its physiological state. This “origin marker” is responsible for their function and confers organotropic properties that give them specificity of action [38,39]. Since exosomes can penetrate tissues inaccessible to platelets, such as joints, lymph and bone marrow, the dissemination of platelet components in tissues and organs beyond the blood may be one of their most important functions. Thus, platelet exosomes have been found to participate in a variety of important biological and pathological processes, including coagulation, angiogenesis, inflammation, immunoregulation and tumor progression [40].

In this sense, human blood derivatives have been proposed as replacements for xenogeneic supplements [8,41]. In recent years, the use of platelet-rich plasma (PRP) has undergone a major development in many applications of the regenerative medicine field [9,16,42,43]. Its rationale for use lies in the physiological role of platelets, which, upon activation, release growth factors and other bioactive molecules, thus promoting the wound healing process [42,43,44,45,46,47,48]. Still, depending on the method to obtain PRP, the composition and concentration of its components may be affected and, ultimately, its biological effect [9,11,48,49,50].

Thus, the aim of this review is to gather the current evidence on the use of PRP as an alternative to the widely used xenogeneic products, based on animal sera, and as a cell culture supplement aimed at cell therapies in order to establish criteria for the optimal characteristics of PRP for this application.

## 2. Methods

**Figure 1:**
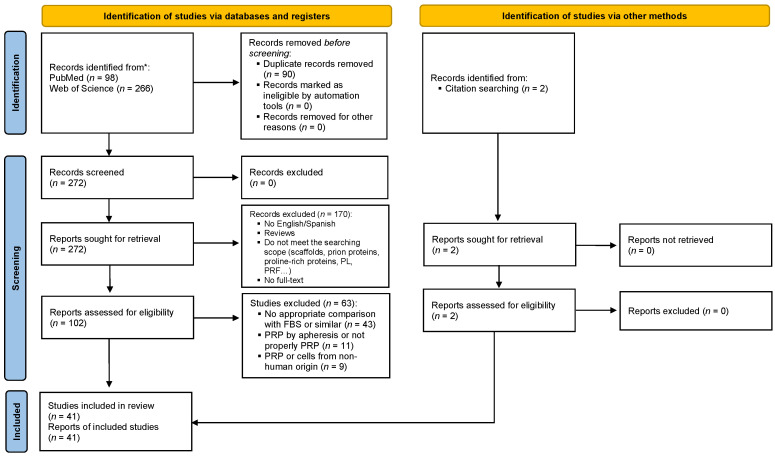
Flowchart summarizing the identification, screening, eligibility and inclusion of the studies in this review following the Preferred Reporting Items for Systematic Reviews and Meta-Analyses (PRISMA) guidelines.

This systematic review was performed following the Preferred Reporting Items for Systematic Reviews and Meta-Analyses (PRISMA) guidelines (Figure 1). It was based on literature searches performed in PubMed and the Web of Science database until 4 June 2021 using ((PRP) OR (platelet rich plasma) OR (plasma rich in growth factors) OR (PRGF)) AND (cell culture) AND (cell culture supplement) as the search strategy. The inclusion criteria were (1) the use of PRP or PRP derivatives as supplement in the culture medium and (2) the presence of a control group (xenogenic supplement). Papers that met the following criteria were excluded from the analysis: (1) the language of the article was any other than English or Spanish; (2) out of scope; (3) non-human origin of PRP or cells; (4) being prepared by aphaeresis or not PRP; (5) clinical studies; (6) reviews, thesis, book chapters or communications at conferences; (7) no full-text available; and (8) duplicates. The protocol of this systematic review was not registered.

Data extraction: The articles in each database were evaluated for inclusion in this review by two independent reviewers (MZ and MT) according to the selection criteria. First, the articles were screened by reading the title and the abstract. Then, the full text of those articles that could be eligible or were doubtful for inclusion were consulted. The reviewers resolved possible discrepancies throughout the entire process by consensus. For data extraction, a template was created as a file in Microsoft Excel to include the following data: author and year of publication, cell phenotype, type of blood-derived supplement, PRP preparation method (type of anticoagulant, number of centrifugations, PRP obtaining and activation method) and composition, xenogeneic product tested, dose of PRP and xenogeneic supplementation in the culture medium, type of assays and results.

Assessment of the reporting quality and risk of bias: The criteria reported by Golbach et al. was applied to assess the quality and the risk of bias [51]. The presence (“yes/partly”) or absence (“no”) of essential information determined the reporting quality of the article. Three grades for the risk of bias were used—low, moderate, or high—depending on whether the answers were “yes”, “partly” or “no”, respectively. If details were not provided, then the risk of bias was judged as unknown.

## 3. Results

The search strategy produced a total of 366 articles to which 2 papers were added from other sources (Figure 1). However, 92 articles were removed as duplicates. Consequently, the number of papers to be more exhaustively screened was 274, of which 170 met the exclusion criteria and were also removed. Finally, 41 papers, as shown in Table 1, were included for the analysis.

### 3.1. Reporting Quality and Risk of Bias

**Figure 2:**
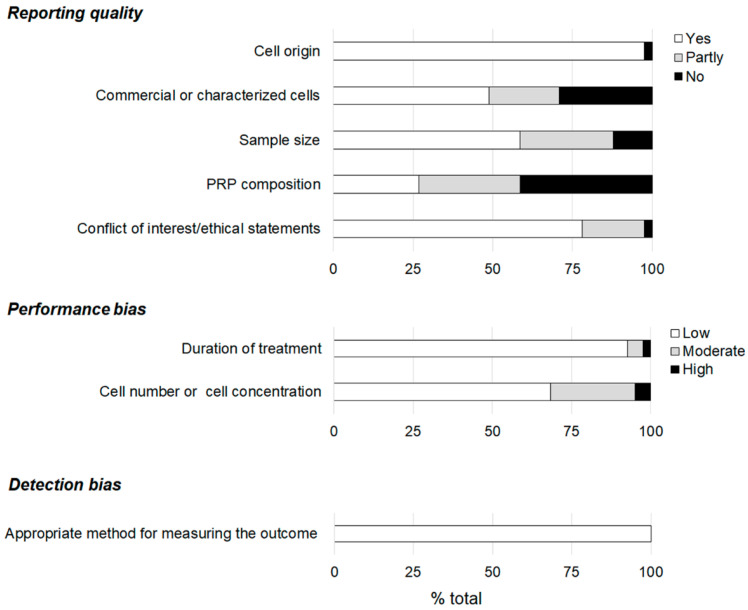
Assessment of the quality and risk of bias of the included studies according to the criteria reported by Golbach et al. [51].

The assessment of the reporting quality indicated that the aspects on which the studies focused the most were cell origin and the statements regarding the conflict of interest or ethical aspects (Figure 2). The composition of the PRP was the least considered, being completely reported in less than 30% of the included articles. Less than 50% of the included studies completely reported on cell characteristics. Sample size was adequately reported in almost 60% of the studies. Low performance bias was judged in most of the articles, and it was more favorable for the item regarding duration of treatment. The method used for measuring the outcomes was considered appropriate in all the reviewed articles.

### 3.2. Human Versus Xenogeneic Cullture Medium Supplement

Regarding the xenogeneic culture medium supplement, FBS (also known as FCS) was the only animal origin supplement used in the reviewed papers. Table 2 describes the methods used for the preparation of the PRP.

A complete description of the cell composition of PRP was provided only in two articles, and commercial systems were used in another nine articles (PRGF [55,80,83], Regenkit [50], Arthrex [70,71,72], GPS III [73] and Harvest SmartPrep System [74]). The platelet concentration was lower than 1 × 10^6^ platelets/µL in 13 studies with a range from 0.141 × 10^6^ to 0.8 × 10^6^ platelets/µL [50,56,57,59,60,62,68,70,71,72,76,77,80]. Most of these studies had a platelet concentration of ≤0.5 × 10^6^ platelets/µL [50,56,57,59,62,70,71,72,76,80]. In 10 studies, the platelet concentration was higher than 1 × 10^6^ platelets/µL (range from 1.086 × 10^6^ to 10 × 10^6^ platelets/µL) [54,57,65,69,70,73,78,79,91,92]. Most of these studies had a concentration between 1 × 10^6^ and 3 × 10^6^ platelets/µL [54,57,65,69,70,73,91,92]. However, there was a consensus among all the studies included in the review in preparing a leukocyte-reduced PRP. Activated PRP versus its inactivated form was the most used option (84%), whereas no information concerning this issue was available in a considerable number of articles (22%). For the activation of the platelets, different agents were used: calcium chloride (31% PRP products), thrombin (34% PRP products) and calcium chloride + thrombin (3% PRP products) (Table 2). Physical methods of activation were also utilized: freezing (24% PRP), freezing + sonication (3% PRP products) and shaking for 9 days (3% PRP products). The PRP or the PRP-derived supernatant were stored frozen at −80 °C, cold at 4 °C, or lyophilized and stored at different temperatures.

#### 3.2.1. Screening for the Optimal Dose of PRP in the Culture Medium

The screening for the optimal dose was not performed in all the included studies. For BM-MSCs, two studies screened the optimum dose of PRP-derived supernatants and a value of 10% was reached [52,67]. However, do Amaral et al. showed similar results to 10% FBS at a PRP concentration of 2.5% rather than 10% [61]. In another study, low concentrations (≤2%) of PRP were screened to prevent culture medium hardening to gel and 0.5% was selected as the optimum dose [68]. In the case of AT-MSCs, there are five studies that screened PRP-derived supernatants as an optimal dose against 10% FBS [54,56,57,71,90]. All these studies agree that a 10% PRP supplement would be the optimal dose. For the same cell phenotype, similar positive results were obtained using a 10% concentration [64,73,78,81], although 20% produced an optimal concentration in another three studies [70,71,72]. In the same way, the non-activated PRP was screened [56,59,72]. To prevent culture medium gelation, Beccia et al. selected a dose of 2%, whereas a dose of 0.5% was chosen by Kishimoto [55]. For Atashi et al., the optimal dose was 20% and did not report any gelling of the culture medium [50], while Rosadi et al. used a concentration of 10% PRP [80]. Only the study of Beccia et al. showed a lower cell proliferation for PRP with respect to the xenogeneic supplement [55]. It is worth mentioning that the use of non-activated PRP was more effective in inducing cell proliferation than PRP-derived supernatants in the study by Atashi et al. [50].

For Wharton’s Jelly-derived MSCs, two experimental works found the optimal concentration of PRP supernatants at 10% [52,54]. In the case of dental pulp stem cells, PRP and human platelet lysate (supernatant) were screened at concentrations of 10% and 20% against 10% of FBS [58]. Both concentrations significantly increased the cell proliferation, with the highest being for the human platelet lysate at 20%. Better results for 10% of PRGF supernatant than 10% FBS were obtained in the isolation, migration, proliferation and differentiation (osteogenesis and adipogenesis) of DPSCs [49]. Similarly, 10% PRP produced the highest proliferation against 2% FCS [83]. Regarding human dermal fibroblasts, Berndt et al. screened different concentrations of PRP (1–50%) and the maximum response in cell proliferation and metabolic activity was reported at a concentration of 20% [56,57]. Xian et al. co-cultured dermal keratinocytes and fibroblasts to compare 5% FBS with 10% and 20% of PRP [90] as culture medium supplements, producing an increased tissue remodeling promoted by 10% PRP and encouraging inflammation and collagen deposition by 20% PRP. Human meniscal fibrochondrocytes showed a higher proliferation with 10 and 20% PRP as compared to 10% FBS, with the mRNA expression of collagen type I + being significantly lower at 3 days, but not at 7 days, for the PRP [62]. Human limbal progenitor cells were cultured with 10% of PRGF supernatant and achieved a faster growth while maintaining the stem/progenitor phenotype in comparison with FBS (5% or 10%) [63,79]. Moreover, in addition to enhancing the colony-forming efficiency, PRGF could provide a fibrin scaffold for culturing human limbal progenitor cells [79]. Regarding the stromal vascular fraction, 10% of t-PRP enhanced the total number of cells without altering their clonogenicity as compared to 10% FBS [65,87]. Kazemnejad et al. compared different blood derivatives for the culture of human menstrual-blood-derived stem cells [66]. They found a similar effect for 10% PRP and 10% FBS on promoting the cell proliferation and mineralization process. However, osteogenic markers, such as osteocalcin and alkaline phosphatase, were higher in cells cultured with 15% PRP and 15% platelet gel supernatant compared to 15% FBS, respectively, although the human platelet releasate (no plasma proteins) was the one that scored more differences with FBS. Regarding periodontal ligament cells, 5% PRP, 10% PRP and 5% PPP enhanced more cell proliferation than 10% FBS [73]. Okada et al. observed that 10% and 20% of PRGF supernatants increased human dental follicle cells proliferation in comparison to 10% FBS and upregulated the gene expression related to bone regeneration [76]. Furthermore, the cell proliferation of ectomesenchymal stem cells from human exfoliated teeth was reported to be more enhanced after being cultured with 10% PRP (without growth factor supplements) than the results that were obtained with 2% FCS (supplemented with growth factors) [82]. However, at higher passages, it induced changes in the cell phenotype. Talebi et al. reported that CCRF-CEM proliferation increased at 10% and 15% PRP as compared to 10% FBS [85]. Human umbilical-cord-blood-derived MSCs cultured with different concentrations of PRP achieved the highest proliferation with 10% PRP [88]. Moreover, in other cell phenotypes (human ovarian cells and human gingival fibroblasts), better results were obtained by adding 10% PRP to the culture medium, as compared to 10% FBS [64,78]. Finally, human articular chondrocytes also showed a higher proliferation with 5% regenerated freeze-dried PRP compared to 10% FCS [75].

Most of the reviewed articles included stem cells, since those that assessed the xenogeneic supplement substitution for PRP in completely differentiated primary cells were only about a quarter. With regard to the origin of the cell type assessed, despite finding a high variability, cells from the adipose tissue were the most analyzed phenotype (17 out of the 41 articles), followed by those from dental origin. However, there was a high number of articles where minority phenotypes were found and, on the contrary, others in which cells from different origins were included (Table 1). Nonetheless, in order to make the comprehension of the results easier, the assessment of xenogeneic supplement substitution for PRP was performed according to the cell phenotype origin instead of referring to each revised article.

#### 3.2.2. Adipose Tissue

Most of the studies included in this section evaluated the replacement of FBS/FCS at 10 or 20% concentrations. Although the PRP was screened over a wider range of concentrations, most of the articles (94%) did show that PRP can replace the xenogeneic culture supplements.

In all [50,52,53,55,60,65,67,68,69,70,71,72,74,77,80,86] but one paper [87], the ability of PRP to replace FBS was assessed in cell proliferation, cell growth or cell cycle processes. All authors [50,52,53,60,65,67,68,69,70,71,72,74,77,80,86] except Beccia et al. [55] showed similar or even statistically superior results of PRP when compared to FBS. Some of them pointed out that the cell response to PRP was dose-dependent [56,57,75,76,90]. However, there was a disparity of conclusions regarding the percentage that stimulates the highest proliferation rate. Amable et al. [52] claimed that PRP inhibits cell growth above 10%. In addition, Atashi et al. [50] reported less effective results in the case of activated PRP (tPRP) compared to non-activated PRP (nPRP), although in both cases they were superior to FBS. Lang et al. [70] and Lobil et al. [72] went even further by determining the mechanisms underlying PRP stimulation of proliferation. This effect could be mediated by the inactivation of Phosphatidylinositol 3,4,5-trisphosphate 3-phosphatase (PTEN) that might then activate the PKB/AKT pathway. In fact, Chieregato et al. [60] reported the involvement of other signaling pathways, such as MEK-1/2. As it was mentioned previously, there was one paper whose conclusions were the opposite [55]. That is, PRP promoted cell proliferation, but at significantly lower level than FBS. This, however, could be related to the lower tested concentration of PRP. In this case, PRP was applied at 2%, whereas FBS was applied at 10%.

Cell morphology was also the subject of study in five studies [59,64,73,78,81]. All articles but Beccia et al. [55] agreed that the cells cultured with PRP were smaller and more spindle-shaped than those cultured with the xenogeneic supplements. The cell phenotype remained unchanged regardless of the supplement used [56,64,71,73]. Moreover, the use of PRP reduced cellular senescence [77] and did not alter chromosomal stability as revealed by cytogenetic analysis [50]. The colony-forming capacity of these stem cells was also evaluated [64,69,73,81], with the clonogenicity with both supplements not being different. In fact, Chieregato et al. [60] and Phetfong et al. [77] reported even more colony-forming units when the cells were cultured with PRP, maintaining the self-renewal of human adipose-derived stem cells after long-term culture.

The effect of PRP addition on cell differentiation was also widely studied. Two studies evaluated the trilineage differentiation [50,52]. Atashi et al. [50] observed that the differentiation potential was not affected regardless of the supplement used. In turn, Amable et al. [52] reported different results depending on the type of differentiation. PRP reduced adipogenic capacity, but increased osteogenic and chondrogenic differentiation. Interestingly, these authors also assessed the gene expression of pluripotent, adipogenic, osteogenic and chondrogenic markers. The use of PRP stimulated the expression of pluripotent genes and thereby downregulated differentiation markers, except for one of the quantified osteogenic markers (BMP2). Four studies assessed the effect of the PRP only on osteogenic and adipogenic differentiation [64,71,73,81]. The results showed that human adipose-derived stem cells were able to similarly differentiate with both culture supplements (PRP or FBS). Furthermore, Phetfong et al. [77] demonstrated a more robust osteogenic differentiation in the presence of the PRP. This was in accordance with the results of Chieregato et al. [60]. However, not only did they report an increase in osteogenesis, but also in adipogenic differentiation after cell exposure to PRP. Another two studies assessed only the chondrogenic differentiation and the results were, again, in favor of the use of the PRP supplement [53,80].

Regarding protein synthesis, Kocaoemer et al. showed a slightly higher total protein content in the culture medium of the PRP-treated cells (97.2 mg/mL and 87 mg/mL for tPRP and FCS, respectively) [69]. Moreover, Amable et al. showed that the protein secretion of adipose-tissue derived stem cells was altered by PRP supplementation [52], which is opposite (upregulated or downregulated) to that induced by FBS for most of the analyzed proteins (cytokines, growth factors, extracellular matrix and metalloproteinases). At the genetic level, the use of PRP, instead of FBS, upregulated the expression of BMP-2 and BMP-4 genes, while downregulating the expression of PDGF-B and FGF-2. The genetic expression of TGF-beta and VEGF was not significantly altered.

#### 3.2.3. Oral Tissue

Seven articles were included in this category (Table 1). In all of them, the results were in favor of PRP as a suitable substitute of FBS/FCS to supplement the culture medium. There was no consensus on the optimal concentration of PRP. In fact, different percentages of PRP were studied, while the percentages of FBS/FCS remained unchanged, at 10% or 2%. Only in three of the articles was PRP composition partially detailed or a reference was made to the trademark that was already associated with the pre-defined parameters. Oral cells from different origins were used, but human dental pulp stem cells (hDPSCs) were predominant as they were used in 43% of the articles [55,62,87]. In the remaining studies, human dental follicle cells (hDFCs) [76], gingival fibroblasts [78], ecto-mesenchymal stem cells from human exfoliated deciduous teeth (SHED) [82] and periodontal ligament cells [73] were employed.

The comparison of PRP and FBS in the cell isolation process was only studied by Anitua et al. [49] who reported a significantly higher number of cells per explant in the PRP group.

Cell viability was also evaluated in three articles [62,86,87]. The authors reported that this parameter was not altered by PRP supplementation; in fact, it was comparable to that obtained with the xenogeneic supplement or even higher. Similar results were observed for cell morphology. This remained unchanged in two out of three articles [62,82,86]. Only Ramos-Torrecillas et al. [78] reported that, in the long-term culture (10 passages), two different populations were observed after culturing with PRP, while the culture with FBS yielded only one cell population, which was also corroborated by the antigenic expression of α-actin. These results did not occur in the short-term culture, where the morphology of gingival fibroblasts remained unchanged. The effect of FBS substitution on clonogenic ability was studied in only one article. Martínez et al. [73] reported that PRP stimulated the clonogenic ability of PDL cells. Finally, the effect of FBS substitution on cell senescence and cryopreservation was also evaluated [49]. The authors reported the same behavior for PRP as for the gold standard regarding these two processes.

The effect of replacing xenogeneic supplements with PRP on the stimulation of proliferation was evaluated in all the articles of this category. In the 71% of the studies, cell proliferation was found to be significantly higher in the PRP than in FBS groups. In the remaining 29%, the results were similar for both supplements [82,83]. Anitua et al. [49] and Okada et al. [76] also assessed the effect on cell migration. Both stated an increase after PRP addition, although the differences were only statistically significant in Anitua et al.’s article.

Two studies also evaluated the effect on osteogenic differentiation in hDPSCs and hDFCs [49,76]. The results showed that PRP led to a significant improvement in osteogenesis through an increase in hDPSCs mineralization [49] or through the upregulation of genes such as type I collagen, osteomodulin, alkaline phosphatase, bone morphogenic protein-4 and transforming growth factor-β in hDFCs [76] after culturing with the osteogenic medium supplemented with PRP. However, the adiogenic differentiation of oral cells was assessed in one study. [49] The adipogenesis of hDPSCs was found to be significantly higher in the PRP group than FBS group.

Regarding angiogenesis, Bindal et al. [58] observed that 8 out of the 12 selected pro-angiogenic genes (ANGPT1, EREG, FGF-2, VEGF-A, IGF-1, JAG-1, NPR2 and PLDXC1) were significantly augmented when lipopolysaccharide (LPS)-induced inflamed dental-pulp-derived stem cells (iDPSCs) were treated with 20% PRP-supplemented media [58]. In addition, the expression of genes related to adhesion molecules was also determined. The higher expression of BAI, NRP2, CCL11 and CDH5 and the downregulation of CCl2 and TGFβ3 were observed in 20% of the PRP-treated cells. Cytokine CXCL1, an inducer of microvascular endothelial migration and tube formation in vitro, was significantly expressed in cells treated with 20% PRP compared to FBS. Conversely, the expression of IFNA1 that inhibits angiogenesis during blood vessel remodeling was reported to be significantly increased in the FBS group.

#### 3.2.4. Cartilage Tissue

The possibility of replacing xenogeneic culture supplements with PRP was also evaluated in cartilage cell cultures. Human nasoseptal chondrogenic cells (NCCs) [61], human meniscal fibrochondrocytes (MFCs) [62] and human articular chondrocytes [75] were the phenotypes used in three of the reviewed articles. Different percentages of PRP were also studied from 1% to 20%, all compared to a reference percentage of 10% FBS/FCS. All these studies found that PRP can also replace the xenogeneic cell culture supplement. However, there was also no consensus on the optimal PRP percentage. Do Amaral et al. [61] reported a greater enhancement of chondrogenic phenotype under a 2.5% PRP treatment rather than 10% PRP. In fact, this lower percentage (2.5%) also induced cell proliferation similarly to 10% FBS, while the proliferation rate increased when cells were cultured in 5% and 10% of PRP. These outcomes may in fact be interrelated. Gonzales et al. [62], on the other hand, detected an equal DNA quantity in MFCs cultured in 10% and 20% PRP in comparison with 5% FBS. No differences in collagen type I expression were also detected for these percentages of PRP compared to FBS. So, they concluded that FBS could be replaced by 10% PRP or 20% PRP without altering proliferation and gene expression of human MFCs. Muraglia et al. [75] only tested one percentage of PRP (5%), as they reported, but did not show, that higher concentrations were less effective or even cytotoxic. However, this percentage was enough to stimulate a significantly higher cell proliferation after 6 and 8 days of culture in comparison with 10% FCS.

#### 3.2.5. Bone Marrow Stem Cells (BM-MSCs)

The capability of PRP to expand hMSCs in vitro, comparing to FBS, was tested by Sun et al. in 2008 [84], confirming the similar spindle-shaped fibroblast-like morphology of cells isolated and cultured in media with either of both supplements. No differences regarding the surface markers were described.

Cell proliferation and differentiation were the most analyzed processes. In this sense, the higher proliferation rate sustained by 10% PRP compared to 10% FBS was confirmed [52,84] together with a shorter population doubling time and a lesser amount of early apoptotic cells, while comparable results between both supplements were reported by Kinzebah et al. [67]. What is more remarkable is the increased cell number yielded by the expansion medium supplemented with much lower percentages of PRPs compared to 10% xenogeneic serum [61,68]. Moreover, the effect of different storage conditions was tested by Muraglia et al. [91]; thus, increased MSC colony numbers and average size after culturing with PRP stored at 4 °C or −80 °C and a raised proliferation rate, even if in the case of the regenerated freeze-dried PRP, were described. Regarding the support of BM-MSCs’ differentiation potential, the cells cultured with PRP retained a similar capacity to differentiate towards the osteogenic, chondrogenic and adipogenic lineages in two out of the five experimental works [67,89]. However, other experimental works described a significantly enhanced osteogenic differentiation with a higher ALP activity, increased calcium deposits and bone gene expression promoted by PRP compared to the same percentage of FBS, as well as a reduction in BM-MSCs adipogenic potential [52,84]. Moreover, a higher chondrogenic gene expression and glycosaminoglycans production, when FBS was replaced by PRP in the differentiation medium, producing a higher biochemical and biomechanical improvement, was observed by Do Amaral et al. [61].

#### 3.2.6. Cells from Skin Tissues

Human dermal fibroblasts and keratinocytes are the two skin cell phenotypes used for assessing the xenogeneic culture medium supplement replacement by PRP, with the former being the most analyzed. Regarding the PRP acquisition method, non-activated PRP was used in the majority of the reviewed articles (60%). However, a multitude of favorable arguments were presented for the FBS substitution by PRP regardless of whether PRP is activated or not.

hDFs proliferation assays confirmed the higher promitogenic potential of several PRP percentages versus animal origin supplements [60,61,63,79], showing a dose-dependent increase in proliferation together with an enhancement in cell metabolic activity [56]. Only in one of the reviewed articles were no differences regarding the proliferative effect of PRP compared to FBS reported [90]. Moreover, the hDFs viability increase after 12 days was significantly enhanced by PRP addition comparing to that was obtained using a higher percentage of FBS [59]. In addition, a spindle-shaped morphology through cytoskeleton rearrangements and changes in alpha SMA, and vimentin expression reminding 3D matrix cultures were also described by Berndt et al. [56]. Regarding the effect on the matrix extracellular gene expression, collagen I and III, and fibronectin genes were upregulated when FBS was replaced by PRP, with the collagen differences being statistically significant [90]. When the fibroblast migration rate was assayed, conflicting results showed a decrease in cell motility in the presence of PRP [90], but also a faster beginning of migration in the wound healing assays [56].

With respect to keratinocytes, similar positive outcomes were reported. Thus, an increase in the percentage of proliferating cells along with a fastest cell migration induced by PRP compared to FBS was also described [90].

In addition to all the improvements achieved by the substitution of FBS by PRP being statistically significant, no genomic instability was reported regarding its use.

#### 3.2.7. Umbilical Cord Tissue

Three articles assessed the effect of PRP supplementation on stems cells from the umbilical cord tissue [54,58,93]. Two studies used non-activated PRP, while only one study employed an activated PRP (Table 2).

One study was interested in evaluating the use of aPRP in the isolation of UCB-MSCs in comparison to 10% FBS [88]. The results showed that primary cultures with a complete medium containing 10% aPRP exhibited the highest success, whereas expansion in complete medium containing 5% aPRP was suitable. UCB-MSCs isolated using aPRP maintained their immunophenotypes and multilineage differentiation potential.

Another study evaluated the effect of PRP on the chondrogenic differentiation of hWJ-MSCs [54]. The results showed that 10% PRP was the optimal supplement to support the chondrogenesis of hWJMSCs. It induced the synthesis of the highest quantity of collagen II and also in a faster way. Amable et al. reported that 10% PRP induced the highest proliferation rate and the shortest population doubling time [52]. Wharton’s Jelly-derived mesenchymal stromal cells secreted higher concentrations of chemokines and growth factors than other mesenchymal stromal cells (bone marrow stem cells and adipose tissue-derived stem cells) when cultured in PRP-supplemented media.

#### 3.2.8. Miscellaneous

Two studies used human limbal epithelial stem cells (LESCs) where serum was obtained after the activation and clotting of plasma rich in growth factors (PRGF) [63,79]. Both studies supplemented the medium with 10% sPRGF. However, the FBS dose was different; one study used it at 5% and another study at 10% (Table 1). Hernaez-Moya et al. did not report significant differences regarding the size, stemness and proliferation of genes [63]. However, a lower number of k12 positive cells was observed in cultures maintained with s-PRGF, thus maintaining the stem/progenitor phenotype of LESCs. Riestra et al. observed that sPRGF induced a significantly greater growth area and higher number of cells [79]. Colony-forming efficiency was found to be also higher in the PRGF group. No significant differences in p63-α expression were found.

Hosseini et al. included human ovarian cells in their comparison between PRP and FBS as cell culture supplements [64]. The results showed that PRP better supported the viability and the growth of encapsulated/isolated human primordial and primary follicles. In another study, the PRP effect on the osteogenic differentiation potential of menstrual-blood-derived stem cells (MenSCs) was tested [66]. In this experimental work, FBS was compared with PRP, platelet gel supernatant (PGS) and human platelet releasate (HPR). There was no significant difference between the growth curves of neither the cultured MenSCs in the presence of different human platelet derivatives nor those in FBS. However, osteogenic differentiation was enhanced by PGS, PRP and HPR. Brini et al. compared activated PRGF to FBS in the osteogenic differentiation of human osteoblast cells [59]. The activated PRGF simultaneously enhanced both cell proliferation and osteo-differentiation, suggesting it as a valid alternative to FBS. Using the same cell type, Muraglia et al. compared PRP to FCS [75]. The study showed that PRP enhanced cell proliferation by more than four times.

Talebi showed that human T lymphoblasts from acute lymphoblastic leukemia CCRF-CEM treated with PRP not only were morphologically comparable to those treated by FBS, but also showed a greater viability at the concentrations of 10 and 15% [85]{Talebi, 2021 #3}. PRP supported cell culture, at least in part, via inducing YKL-40 expression at both mRNA and protein levels in a time- and dose-dependent manner.

The enhanced viability and similar proliferation rate of human mesenchymal stem cells (MSCs) of unknown origin, along with typical MSC morphology preservation promoted by PRP supplementation, was reported by Simon et al. [81]. The maintenance of specific antigen expression into the desired ranges, the prevention of cell differentiation and the lack of alteration of the apoptotic and antiapoptotic genes ratio were also observed.

## 4. Discussion

The current systematic review and previously published reviews could be an indicator for the increase in interest in using human PRP as a substitute of xengoneic serum in cell therapy [5,9,10,45,92,93,94]. However, the use of PRP raises several open questions that need to be addressed in order to create standardization in the complex and evolving use of PRP. These questions are related to the definition of the platelet-rich plasma in terms of platelet concentration, leukocyte content, formulation type (activated or non-activated), the activator type where required (calcium ions, thrombin or physical methods) and preparation methods, including the anticoagulant type and concentration.

To answer this question, the PRP preparation needs to be described, so a comparative analysis could be performed. PRP composition was the least considered item, being completely described in less than 30% of the included studies (Table 1). This finding has also been reported by previous systematic reviews [10,94]. Transparency by using a classification system or algorithm that describes the PRP formulations has to be implemented. A similar need, but in the clinical field, has motivated the suggestion of several systems for the classification and standardization of reporting on PRP [95,96,97,98,99]. Based on the results of this review and the classification systems of PRP in the clinical field, several items were identified in order to have a transparent description of PRP from the perspective of cell therapy. These items would allow the comparative analysis and reproduction of PRP by other researchers (Table 3). These items are the features of the blood donor, the medical device for blood extraction, the characteristics of the blood, blood processing for PRP preparation, the definition of the PRP, PRP hallmark, the PRP activation procedure where appropriate, the nomenclature of PRP formulation, the content of key biomolecules, the origin of the PRP relative to the cells, the dose of the PRP and the microbial inactivation where applicable.

This review demonstrated the considerable research that has been dedicated to defining the ideal medium that can substitute xenogenic systems. Platelet-rich plasma is used in different forms (PRP-derived supernatants and non-activated PRP) and at different concentrations. No comparative studies are available to assess the optimal platelet concentration of the PRP for stem cells culturing. Two ranges of platelet concentration could be considered: the first one between 0.14 × 10^6^ and 0.80 × 10^6^ platelets/µL (<1.0 × 10^6^ platelets/µL) [50,56,57,59,60,62,68,70,71,72,76,77,80] and the second between 1.086 × 10^6^ and 10 × 10^6^ platelets/µL (>1.0 × 10^6^ platelets/µL) [54,57,65,69,70,73,78,79,91,92]. However, there is a consensus among all the studies regarding the preparation of a leukocyte-reduced PRP. Cell proliferation is the test that has been widely used to determine the most successful PRP concentration in the culture medium. Thus, the optimal dose of PRP is ranged between 0.5% and 20%, with 10% PRP being the most widely used, which is in agreement with previous systematic reviews [92,93,94]. Regarding platelet activation, the three most common methods include the use of calcium chloride, thrombin and freezing procedure (Table 2). However, there are no comparative studies to recommend an optimal activation method.

Regarding the type of anticoagulant, 23 studies have not specified it. Anticoagulants are commonly used to prevent the coagulation of blood by either neutralizing thrombin (heparin and hirudin) or chelating calcium ions (oxalate, EDTA and citrate) [100,101]. Chelating calcium offers the advantages of recovering the coagulation by adding an excess of calcium ions later. It is important to pay attention to the type and concentration of the anticoagulant in order not to disrupt the size, morphology, counting and activity of the blood cells [102,103]. Even more, changes in cell differentiation and mitogenesis have been reported by altering the concentration of the anticoagulant [104,105]. Sodium citrate is the most common anticoagulant utilized in the reviewed articles (10 studies), being associated with higher platelet recovery and the genetic stability of mesenchymal stromal cells [105].

Pooling and pool size are important parameters to reduce the variability in pooled human platelet lysate regarding the concentration of growth factors and batch-to-batch divergence [106]. However, the potential risk of transmitting diseases may rise as the pool size increases [107]. In this review, 14 studies pooled PRP with a pool size varying between four and nine donors. The most frequent pool sizes were four and eight donors. In two studies, quality control tests were performed [52,67]. Amable et al. assessed the growth factor concentration and compared it with values that had been previously determined [52], while cell proliferation was employed by Kinzebach et al. [67]. In the context of cell-based therapy, the identity (molecular structure/composition, biological, physico-chemical or immunological properties) is a requisite to demonstrate the uniqueness of the raw material [108]. However, this is complicated in the case of platelet-rich plasma as there is no consensus on the optimal platelet concentration and, thus, the concentration of biomolecules. However, all the studies in the review used leukocyte-reduced PRP. For routine use, chemically defined media are necessary for the standardized culturing of MSCs under GMP guidelines [93]. There is a need for more comparative studies under a GMP-compliant manufacturing process using PRP to define the composition criteria that need to be fulfilled. Another possibility is the implementation of performance testing regarding contamination, total proteins, pH and osmolarity [108]. From the standpoint of good manufacturing practice (GMP), PRP should be free from contamination risk, non-immunogenic, non-oncogenic, effective in increasing the cell proliferation rate and effective in retaining unmodified the MSC phenotype and their differentiation capacity [108]. The results of this review support the use of PRP as it was reported to be effective in increasing, or at least not lowering, the cell proliferation rate, maintaining unmodified the MSC phenotype (except for ectomesenchymal stem cells from human exfoliated teeth at higher passages), preserving their genetic stability and supporting their differentiation capacity (Table 2). Moreover, PRP or PRP-derived supernatants have a positive effect on BM-MSCs culturing and also on MSC colony numbers and average size [75], cell proliferation [54,71,72,79,88] and osteogenic [52,84] and chondrogenic differentiation [52], but inhibit adipogenic differentiation [52,84]. In AT-MSCs, PRP treatment promotes cell proliferation [50,52,53,60,67,68,69,70,71,72,74,77,80,86] and chondrogenic [54,56,57,64,78,81,84], osteogenic [54,56,64,73,78,81] and adipogenic differentiation [56,64,73,78,81]. However, studies showed a lower osteogenic differentiation capacity [86], lower proliferation [55] and lower adipogenic differentiation capacity [52] in this cell phenotype. In the case of WJ-MSC culturing, the PRP or PRP-derived supernatants have a positive effect on cell proliferation [52,54] and osteogenic [52] and chondrogenic differentiation [52,54], but inhibit adipogenic differentiation [52]. Similar results have been observed for UCB-MSCs [88]. The PRP supernatant enhanced HDPS cell isolation, migration, proliferation and osteogenic and adipogenic differentiation, but without altering the adipogenic differentiation, senescence or their cryopreservation [49].

However, PRP induced molecular differences in cell culturing when compared to FBS. In stem cells from adipose tissues, Lang et al. [70] and Lobil et al. [72] suggested that enhanced cell proliferation by the PRP could be mediated by the inactivation of PTEN that might then activate the PKB/AKT pathway. Chieregato et al. [60] reported the involvement of other signaling pathways, such as MEK-1/2. Furthermore, Amable et al. [52] showed that the use of PRP stimulated the expression of pluripotent genes and thereby downregulated differentiation markers, except for one of the quantified osteogenic markers (BMP2). Regarding protein synthesis, Kocaoemer et al. showed a slightly higher total protein content in the culture medium of the PRP-treated cells [69]. Moreover, Amable et al. showed that the protein secretion of adipose-tissue-derived stem cells was altered by PRP supplementation [52], being opposite direction (upregulated or downregulated) to that induced by FBS for most of the analyzed proteins (cytokines, growth factors, extracellular matrix and metalloproteinases). At the genetic level, the use of PRP, instead of FBS, upregulated the expression of BMP-2 and BMP-4 genes, while downregulating the expression of PDGF-B and FGF-2. The genetic expression of TGF-beta and VEGF was not significantly altered [52]. In gingival fibroblasts, the use of PRP in long-term culture (10 passages) resulted in changes in cell population (two different posulations in PRP vs one population in the FBS) as corroborated by the antigenic expression of α-actin [78]. These differences were not observed in the short-term culture where the morphology of gingival fibroblasts remained unchanged. For hDPSCs, the use of PRP led to a significant improvement in osteogenesis through an increase in hDPSC mineralization [49] or through the upregulation of genes such as type I collagen, osteomodulin, alkaline phosphatase, bone morphogenic protein-4 and transforming growth factor-β in hDFCs [76]. In an inflammatory model, Bindal et al. [58] observed that 8 out of 12 selected pro-angiogenic genes (ANGPT1, EREG, FGF-2, VEGF-A, IGF-1, JAG-1, NPR2 and PLDXC1) were significantly augmented when lipopolysaccharide (LPS)-induced inflamed dental pulp-derived stem cells (iDPSCs) were treated with 20% PRP-supplemented media [58]. The higher expression of BAI, NRP2, CCL11 and CDH5 and the downregulation of CCl2 and TGFβ3 were observed in 20% of the PRP-treated cells. Cytokine CXCL1, an inducer of microvascular endothelial migration and tube formation in vitro, was significantly expressed in cells treated with 20% PRP compared to FBS. Conversely, the expression of IFNA1 that inhibits angiogenesis during blood vessel remodeling was significantly increased in the FBS group. In BM-MSCs, the use of PRP led to a significantly enhanced osteogenic differentiation with higher ALP activity, increased calcium deposits and bone gene expression [52,84]. Moreover, a higher chondrogenic gene expression and glycosaminoglycan production was observed, resulting in a higher biochemical and biomechanical improvement [61]. For Wharton’s Jelly-derived stem cells, the use of PRP induced the synthesis of the highest quantity of collagen II and also in a faster way. Amable et al. observed that mesenchymal stromal cells secreted higher concentrations of chemokines and growth factors than other mesenchymal stromal cells (bone marrow stem cells and adipose-tissue-derived stem cells) when cultured in PRP-supplemented media [52].

In a previous systematic review of pre-clinical studies, the combination of PRP and stem cells improved osteogenic and cartilage regeneration, but conflicting results have been reported for periodontal regeneration [10]. Guiotto et al. performed a systematic review similar to the one reported in this article [94]. Improved cell proliferation and differentiation supported the use of human platelet lysate as an alternative to xenogenic sera [92,93,94]. However, that systematic review lacks the assessment of risk of bias and was limited to human plasma lysate.

This review has several limitations. Firstly, there is a lack of complete descriptions of the preparation and characterization of PRP. Furthermore, there is an absence of comparative studies among different PRP. All these limitations make it difficult to derive recommendations regarding PRP characteristics for the culture of primary cells regarding cell therapies.

## 5. Conclusions

The use of leukocyte-depleted PRP as an alternative to xenogeneic sera for the culturing of stem cells was demonstrated to be feasible by this review. However, there is a need to improve the description of the PRP preparation methodology as well as its composition. Furthermore, there is a need to establish a potency/performance test and comparative studies among different PRP compositions to determine quality control parameters and universally accepted guidelines.

## Figures and Tables

**Table 1:** Detailed information of the studies included in this review.

Reference	Phenotype	Comparison Groups	Assays	Conclusion
Amable et al., 2014 [52]	BM-MSC, AT-MSCs and WJ-MSC	10% FBS vs. 1%, 2.5%, 5%, 10%, 20%, 30%, 40%, 50% PRP	Proliferation, trilineage differentiation, gene expression, and cytokine, growth factor and extracellular matrix quantification	✓
Anitua et al., 2019 [49]	hDPSCs	10% FBS vs. 10% PRGF	Isolation, migration, proliferation, osteogenic and adipogenic differentiation, senescence and cryopreservation	✓
Atashi et al., 2015 [50]	AT-MSCs	10% FBS vs. 1%, 5%, 10%, 20%, 40%, 60% of either nPRP or tPRP	Cell viability, cell proliferation, cell phenotype, trilineage differentiation, chromosome stability cytogenetic analysis	✓
Barlian et al., 2018 [53]	ADSCs	10% FBS (control) vs. 5%, 10%, 20% PRP	Chondrogenic differentiation	✓
Barlian et al., 2020 [54]	WJ-MSCs	10% FBS vs. 10% PRP	Chondrogenic differentiation (collagen type II, GAG accumulation).	✓
Beccia et al., 2021 [55]	ASCs	10% FBS vs. 2% PRP	Morphology and proliferation	✕
Berndt et al., 2019 [56]	NHDF	10% FBS vs. 1%, 5%, 10%, 20%, 30%, 40%, 50% PRP	Cell proliferation, cell cycle analysis, cell morphology, alpha-SMA and vimentin expression, metabolic activity assessment, cell adhesion, wound healing, genomic stability	✓
Berndt et al., 2021 [57]	NHDF	10% FBS vs. 1%, 5%, 10%, 20%, 30%, 40%, 50% PRP	NHDF proliferation and activation.	✓
Bindal et al., 2019 [58]	hiDPSCs	10% FBS vs. 10%, 20% PRP	Viability, proliferation, proangiogenic gene expression, proangiogenic growth factor release	✓
Brini et al., 2016 [59]	hDFs / hObs	10% FBS vs. 5% PRGF (cell proliferation and viability) 10% FBS vs. 2.5% PRGF (osteoblast differentiation)	Cell proliferation and viability, osteogenic differentiation	✓
Chieregato et al., 2011 [60]	ADSCs	10% FBS vs. 10% hPRP	Morphology, CFU, proliferation and MEK-1/2 role, multiple differentiation capacity, immunophenotype	✓
do Amaral et al., 2015 [61]	NCCs and MSCs from bone marrow	10% FBS vs. 1%, 2.5%, 5%, 10% PRPr	Cell proliferation, GAGs, pellet area measurement, chondrogenic genes quantification, sGAG quantification	✓
Gonzales et al., 2013 [62]	MFCs	10% FBS vs. 5%, 10%, 20% PRP	DNA quantification, gene expression (col I, col II and aggrecan), histology (H&E)	✓
Hernáez-Moya et al., 2020 [63]	LESCs	5% FBS vs. 10% s-PRGF	Cell growth, cell size and gene expression of stem/progenitor limbal cells markers and K12 marker for corneal epithelial differentiation	✓
Hosseini et al., 2017 [64]	Human ovarian cells	10% FBS vs. 10% PRP	Follicle growth and viability assessment. Histological analysis.	✓
Ismail et al., 2020 [65]	SVF cells	10% FBS vs. 10% tPRP	Cell number and clonogenicity	✓
Kazemneja et al., 2014 [66]	MenSCs	10% FBS vs. PGS vs. PRP vs. HPR (proliferation assays) 15% FBS vs. PGS vs. PRP vs. HPR (differentiation assays)	Cell proliferation and osteogenic differentiation (Alizarin Red, ALP activity, OCN level)	✓
Kinzebach et al., 2013 [67]	LA-MSC and BM-MSC	2.5%, 5%, 7.5%, 10% FBS vs. 2.5%, 5%, 7.5%, 10% tPRP	Expansion of MSCs and differential proteomics, proliferation and stimulation assays. Growth factors quantification and cytokine receptors expression. Adipo- and osteogenic differentiation.	✓
Kishimoto et al., 2013 [68]	ASCs and BMSCs	0.125%, 0.25%, 0.5%, 1%, 2%, 4% FBS vs. 0.125%, 0.25%, 0.5%, 1%, 2%, 4% PRP (optimal concentration) 2%, 10% FBS vs. 0.5%PRP (proliferation)	Determination of the supplement optimal concentration, proliferation	✓
Kokaoemer et al., 2007 [69]	AT-MSCs	10% FCS vs. 10% tPRP	Morphology, adhesion, CFU, cumulative population doubling rates, adipogenic and osteogenic differentiation, immunophenotype	✓
Lang et al., 2017 [70]	ASCs	20% FCS vs. 10%, 20% PRP	Cell cycle analysis, expression of PDGF receptorβ, c-MYC, and MEK-1, PDGF receptor β Inhibition	✓
Loibl et al., 2016 [71]	ASCs	20% FCS vs. 10%, 20% ACS	Cell cycle analysis	✓
Loibl et al., 2016 [72]	ASCs	20% FCS vs. 10%, 20% ACS	Cell cycle analysis and proteomic profile	✓
Martínez et al., 2019 [73]	PDL cells	10% FBS vs. 2.5%, 5%,10% PRP	Cell proliferation and clonogenic proliferation	✓
McLaughlin et al., 2016 [74]	ASCs	10% FBS vs. 10% tPR	Morphology, growth rate, gene expression (BMP-2, BMP-4, VEGF, TGF-beta, PDGF-B and FGF-2)	✓
Muraglia et al., 2014 [75]	MSCs from bone marrow/human skin fibroblasts/hObs/human articular chondrocyte	10% FCS vs. 5% PRP	Clonogenic assay (MSCs) and cell viability of primary cultures	✓
Okada et al., 2016 [76]	hDFCs	10% FBS vs. 1%, 5%, 10%, 20% PRGF	Osteogenic differentiation, cell proliferation, cell migration and osteogenic gene expression	✓
Phetfong et al., 2017 [77]	ADMSCs	FBS vs. Hplasma	Cell morphology, proliferation, CFU, immnuphenotyping, osteogenic and adipogenic differentiation, senescence	✓
Ramos-Torrecillas et al., 2014 [78]	Human gingival fibroblasts	10% FBS vs. 10% PRP	Cell growth rate, cell morphology and antigenic expression	✓
Riestra et al., 2017 [79]	LESCs	10% FBS vs. 10% PRGF	Measurement of the extent of outgrowths of cultures of LEPCs, number of cells and colony forming efficiency. Morphological analysis and immunocytochemistry and quantification of p63-α HLCE.	✓
Rosadi et al., 2019 [80]	ADSCs	10% FBS vs. 10%PRP	Cell proliferation, differentiation assays (GAG levels and mineralization, secretion of TGF-β1, expression of specific stem cell surface protein markers, gene expression)	✓
Simon et al., 2018 [81]	MSCs	10% FCS vs. 10% FCS+bFGF vs. 10% PRP	Cell proliferation, lineage-specific markers, gene expression	✓
Suchánek et al., 2016 [82]	SHED	2% FCS + GFs (FCS+) vs. 2% PRP + GFs (PRP+)	Proliferative capacity, cumulative population doubling, morphology, viability, expressing cluster of differentiation	✓
Suchánková et al., 2014 [83]	hDPSC	2% FCS vs. 2% PRP	Proliferation, population doublings, viability, phenotypic analysis	✓
Sun, Xiaojiang et al., 2008 [84]	MSCs from bone marrow	10% FBS vs. 10% APM	Cell morphology, proliferation, surface markers, growth cycle, and apoptosis; osteogenic differentiation; number and area of ALP^+^CFU-Fs; adipogenic differentiation.	✓
Talebi et al., 2021 [85]	CCRF-CEM	10% FBS vs. 2%, 5%, 10%, 15% PRP	Cell viability and YKL-40 mRNA and protein levels.	✓
Tavakolinejad et al., 2014 [86]	ADSCs	10% FBS vs. 10%, 15% hPRP	Proliferation and osteogenesis	✓
Tchang et al., 2017 [87]	SVF cells	10% FBS vs. 10% tPRP	2D mineralization assay and 3D angiogenesis	✓
Van Pham et al., 2014 [88]	UCB-MSCs	10% FBS vs. 2, 5, 7, 10% PRP	Number of adherent cells and their expansion, percentage of successfully isolated cells in the primary culture, surface marker expression, in vitro differentiation potential following expansion	✓
Vogel et al., 2006 [89]	BM-MSC	2% FCS vs. 3% PRP	Growth rate; osteogenic, adipogenic and chondrogenic differentiation capacity	✓
Xian et al., 2015 [90]	Human keratinocytes and fibroblasts from skin	5% FBS vs. 10%, 20% PRP	Extracellular matrix gene expression, proliferation, migratory property, soluble factors secretion	✓

**ACS**: autologous conditioned plasma; **ADMSCs**: human adipose-derived mesenchymal stem cells; **ADSCs**: human adipose-derived mesenchymal stem cells; **ADSC-SS**: ADSCs cultured on the scaffold; **ALP**: alkaline phosphatase; **ALP^+^CFU-Fs**: alkaline phosphatase–positive (ALP^+^) fibroblast colony-forming units (CFU-Fs) under osteogenic conditions; **APM**: autologous plasma derived from bone marrow; **ASCs**: human adipose-derived stem cells; **AT-MSCs**: human adipose-derived mesenchymal stem cells; **BM-MSC**: human mesenchymal stromal cells from bone marrow; **BMP-2**: bone morphogenetic protein 2; **BMP-4**: bone morphogenetic protein 4; **BMSCs**: bone marrow-derived mesenchymal stem cells; **CCRF-CEM**: human T lymphoblasts from acute lymphoblastic leukemia; **CFE**: colony-forming efficiency; **CFU**: colony-forming units; **FBS**: fetal bovine serum; **FCS**: fetal calf serum; **FGF-2**: Fibroblast growth factor 2 (basic); **GAG:** glycosaminoglycans; **hDFs**: human dermal fibroblasts; **hDFCs**: human dental follicle cells; **hDPSCs**: human dental pulp stem cells; **hiDPSCs**: human inflamed dental pulp stem cells; **HAS**: human serum albumin; **HPR**: human platelet releasate; **hObs**: human osteoblasts; **Hplasma**: human plasma; **hPRP**: human platelet-rich plasma; **LA-MSC**: lipoaspirate-derived MSC; **LESCs**: Human limbal epithelial stem/progenitor cells; **MenSCs**: menstrual-blood-derived stem cells; **MFCs**: human meniscal fibrochondrocytes; **MSCs**: mesenchymal stem cells; **NCCs**: human nasoseptal chondrogenic cells; **NHDF**: Normal human dermal fibroblasts; **nPRP**: non-activated PRP; **OCN**: osteocalcin; **OIM**: osteogenic induction medium; **PDGF-B**: platelet-derived growth factor subunit B; **PDL**: periodontal ligament; **PGS**: platelet gel supernatant; **PRGF**: plasma rich in growth factors; **PRPr**: platelet-rich plasma releasate; **TGF-beta**: transforming growth factor beta; **SHED**: ecto-mesenchymal stem cells from human exfoliated deciduous teeth; **s-PRGF**: Serum derived from plasma rich in growth factors; **SVF**: stromal vascular fraction; **tPRP**: thrombin-activated PRP; **UCB-MSCs**: human umbilical--cord blood-derived MSCs; **VEGF**: vascular endothelial growth factor; **WJ-MSC**: Wharton’s Jelly-derived MSC; **✓**: experimental results supporting the replacement of xenogeneic supplements with PRP; **✕**: best experimental results for the FBS supplement.

**Table 2:** Description of the PRP acquisition process in the reviewed articles.

Reference	Type of Anticoagulant	Comparison Groups	Number of Centrifugations	PRP Acquisition	Activation Method
Amable et al., 2014 [52]	ACD	10% FBS vs. 1%, 2.5%, 5%, 10%, 20%, 30%, 40%, 50% PRP	Two	Platelet-containing plasma above the buffy coat. Platelets concentrated and suspended in a smaller volume of plasma.	Calcium chloride
Anitua et al., 2019 [49]	Sodium citrate	10% FBS vs. 10% PRGF	One	PRGF: plasma column just above the buffy coat.	Calcium chloride
Atashi et al., 2015 [50]	Sodium citrate	10% FBS vs. 1%, 5%, 10%, 20%, 40%, 60% of either nPRP or tPRP	One	Regenkit: plasma containing platelets above the white and red blood cells.	nPRP: non-activated
	W/o	10% FBS (control) vs. 5%, 10%, 20% PRP		Regenkit: plasma over the red and most of the white blood cells formed a clot. The serum extracted from the clot was added 1:10 to PRP to activate the platelets and obtain tPRP.	tPRP: thrombin
Barlian et al., 2018 [53]	NA	10% FBS vs. 10% PRP	NA	NA	NA
Barlian et al., 2020 [54]	NA	10% FBS vs. 2% PRP	NA	NA	NA
Beccia et al., 2021 [55]	Buffered solution of sodium citrate, theophylline, adenosine and dipyridamole	10% FBS vs. 1%, 5%, 10%, 20%, 30%, 40%, 50% PRP	Two	Plasma portion separated from cells.	Non-activated
Berndt et al., 2019 [56]	NA	10% FBS vs. 1%, 5%, 10%, 20%, 30%, 40%, 50% PRP	One	Plasma containing platelets, remained above the gel layer, was homogenized by turning the tube five times.	Non-activated
Berndt et al., 2021 [57]	NA	10% FBS vs. 10%, 20% PRP	One	The red and white blood cells are trapped under the gel, and platelets settled on the surface of the gel are resuspended by inverting the tube.	Non-activated
Bindal et al., 2019 [58]	NA	10% FBS vs. 5% PRGF (cell proliferation and viability) 10% FBS vs. 2.5% PRGF (osteoblasts differentiation)	NA	NA	Freezing
Brini et al., 2016 [59]	Sodium citrate	10% FBS vs. 10% hPRP	One	PRGF, the 2 mL plasma just above the buffy coat containing the highest platelets concentration, was collected.	Calcium chloride
Chieregato et al., 2011 [60]	Heparin	10% FBS vs. 1%, 2.5%, 5%, 10% PRPr	One	NA	Freezing and sonication
Do Amaral et al., 2015 [61]	Citrate sodium	10% FBS vs. 5%, 10%, 20% PRP	Two	PRP from protocol 2: The upper plasma fraction without leukocyte and red cells was centrifuged. Platelets pellet was resuspended with the supernatant (platelet-poor plasma).	Calcium chloride
Gonzales et al., 2013 [62]	Hirudin	5% FBS vs. 10% s-PRGF	One	The upper phase containing PRP.	Thrombin??
Hernáez-Moya et al., 2020 [63]	Sodium citrate	10% FBS vs. 10% PRP	One	The complete supernatant fraction without red and white blood cells.	Calcium chloride
Hosseini et al., 2017 [64]	Acid citrate solution	10% FBS vs. 10% tPRP	Two	The top and middle layers after the first centrifugation were centrifuged again and the remaining 0.5 mL of plasma containing precipitated platelets was mixed evenly and considered to be PRP.	Thrombin
Ismail et al., 2020 [65]	NA	10% FBS vs. 10% PGS vs. 10% PRP vs. 10% HPR (proliferation assays) 15% FBS vs. 15% PGS vs. 15% PRP vs. 15% HPR (differentiation assays)	NA	Two platelet concentrates from buffy coats extracted from whole-blood donations of four AB blood group-typed donors were pooled and suspended in the plasma of one AB donor.	Thrombin
Kazemnejad et al., 2014 [66]	NA	2.5%, 5%, 7.5%, 10% FBS vs. 2.5%, 5%, 7.5%, 10% tPRP	NA	NA	PRP: freezing PGS: thrombinHPR: thrombin
Kinzebach et al., 2013 [67]	NA	0.125%, 0.25%, 0.5%, 1%, 2%, 4% FBS vs. 0.125%, 0.25%, 0.5%, 1%, 2%, 4% PRP (optimal concentration) 2%, 10% FBS vs. 0.5% PRP (proliferation)	NA	Buffy coat-derived pooled platelet concentrates.	Thrombin
Kishimoto et al., 2013 [68]	Sodium citrate	10% FCS vs. 10% tPRP	Two	The upper 1 cm of the erythrocyte layer was collected as the PRP layer.	Freezing
Kokaoemer et al., 2007 [69]	NA	20% FCS vs. 10%, 20% PRP	NA	Pooled platelet concentrate out of buffy coats.	Thrombin
Lang et al., 2017 [70]	W/o	20% FCS vs. 10%, 20% ACS	One	Arthrex: a plasma layer appeared on the top and the red/white blood cell layer was apparent at the bottom. The plasma containing the platelets was isolated.	Freezing
Loibl et al., 2016 [72]	NA	20% FCS vs. 10%, 20% ACS	One	Arthrex	NA
Loibl et al., 2016 [71]	NA	10% FBS vs. 2.5%, 5%, 10% PRP	One	Arthrex: a plasma layer appeared on the top and the red/white blood cell layer was apparent on the bottom. The plasma, containing the platelets, was isolated.	Freezing
Martínez et al., 2019 [73]	NA	10% FBS vs. 10% tPR	One	GPS III	Calcium chloride and thrombin
McLaughlin et al., 2016 [74]	NA	10% FCS vs. 5% PRP	NA	Harvest SmartPrep System	Thrombin
Muraglia et al., 2014 [75]	NA	10% FBS vs. 1%, 5%, 10%, 20% PRGF	Multiple	Buffy coat samples. The platelet pellet was brought to a final volume with PPP to obtain a concentration of 10 × 10^6^ platelets/µL.	Freezing
Okada et al., 2016 [76]	Sodium citrate	10% FBS vs. 10% Hplasma	One	PRGF: the plasma fraction (1 mL over the buffy coat) was collected as F2.	Calcium chloride
Pham et al., 2013 [88]	NA	10% FBS vs. 10% PRP	Two	After the second centrifugation, the platelet pellet was resuspended with the third of the plasma volume.	Calcium chloride
Phetfong et al., 2017 [77]	NA	10% FBS vs. 10% PRGF	NA	It was prepared from FFP.	Calcium chloride
Ramos-Torrecillas et al., 2014 [78]	Lithium heparin	10% FBS vs. 10% PRP	Two	The whole plasma portion and top layer of red blood cells. After a new centrifugation, the upper portion of the plasma was discarded, and the remainder was the PRP.	NA
Riestra et al., 2017 [79]	Sodium citrate	10% FCS vs. 10% FCS + bFGF vs. 10% PRP	One	Liquid PRGF: the lower 2 mL of the plasma column (F2) was discarded. The rest of the plasma column (F1) was drawn off avoiding the buffy coat.	Calcium chloride
Rosadi et al., 2019 [80]	NA	2% FCS + GFs (FCS+) vs. 2% PRP + GFs (PRP+)	NA	NA	NA
Simon et al., 2018 [81]	NA	2% FCS vs. 2% PRP	Two	The platelet-rich layer above the buffy coat was centrifuged and the resulting platelet pellet was resuspended to obtain PRP.	Non-activated
Suchánek et al., 2016 [82]	NA	10% FBS vs. 10% APM	NA	NA	NA
Suchánková et al., 2014 [83]	NA	10% FBS vs. 2%, 5%, 10%, 15% PRP	NA	NA	NA
Sun, Xiaojiang et al., 2008 [84]	NA	10% FBS vs. 10%, 15% hPRP	Two	Bone marrow was concentrated through density gradient centrifugation. After removing the remaining red blood cells and fatty droplets by centrifugation, the APM was collected.	NA
Talebi et al., 2021 [85]	Sodium citrate	10% FBS vs. 10% tPRP	Two	Supernatant from the first centrifugation, including PRP, was again centrifuged and the lower half part, which contains a large number of platelets in the form of the platelet plug, was considered as PRP.	Shaking at 22 °C for nine days.
Tavakolinejad et al., 2014 [86]	NA	10% FBS vs. 2%, 5%, 7%, 10% PRP	Two	The platelets were precipitated and the plasma was removed; then, the platelets were resuspended in 50 mL plasma	Freezing
Tchang et al., 2017 [87]	NA	2% FCS vs. 3% PRP	NA	Platelet concentrates from buffy coats extracted from whole blood.	Thrombin
Vogel et al., 2006 [89]	Citrate–phosphate–dextrose	5% FBS vs. 10%, 20% PRP	One	Allogenic leukocyte-depleted PRP was obtained from a blood bank. Pooled buffy coats were centrifuged and leukocyte-depleted by filtration.	NA
Xian et al., 2015 [90]	Sodium citrate	5% FBS vs. 10%, 20% PRP	Two	Harvested PRP without red cells was concentrated by discarding 2 mL of plasma after the second centrifugation.	Non-activated

**ACD:** citrate dextrose solution A; **APM:** autologous plasma derived from bone marrow; **F2:** fraction 2; **FFP:** fresh frozen plasma; **HPR:** human platelet releasate; **NA:** not available; **nPRP:** non-activated PRP; **PGS:** platelet gel supernatant; **PPP:** platelet-poor plasma; **PRGF**: plasma rich in growth factors; **PRP:** platelet-rich plasma; **tPRP:** thrombin-activated PRP; **W/o:** without.

**Table 3:** Items for the transparent description of PRPs in research for cell therapy applications.

Item	Description
Blood donor features	Age, sex and systemic health status (ASA)
Medical device for blood extraction	Type of medical devise (bags, vacutainer, syringe) and commercial information. Type of additives and their concentration Blood to anticoagulant ratio
Blood characteristics	Pooled or individually processed Hematocrit and concentration of platelets, leukocytes and red blood cells (RBCs) Blood group (ABO and Rh systems)
Blood processing for PRP preparation	Procedure: centrifugation, aphaeresis, microfluidic system Equipment used: commercial information Processing parameters: number of cycles, centrifugation force and time
PRP definition	Which specific part of the fractioned blood (plasma and/or buffy coat) is considered PRP?
PRP hallmark	Pooled or individually characterized Volume Concentration of platelets, leukocytes and RBCs Post-processing of PRP (lyophilization and freezing) Use of additives (type and concentration) Storage conditions
Activation	Yes/no Method of activation: calcium ions, thrombin, light, agitation Concentration of activator solution and ratio to PRP
Nomenclature of PRP formulation	Non-activated PRP Activated liquid PRP PRP serum PRP fibrin
Storage conditions	Lyophilized or not Temperature
Key biomolecules content	Biomolecules identification and kits used for quantifications
Origin of PRP relative to the cells	Autologous, allogenic or xenogenic
Pathogen detection	Yes/No; if yes, specify microorganism and the assay used
Microbial inactivation	Yes/No; if yes, specify the procedure
Dose of PRP	Percentage to the volume of cell culture medium

## Data Availability

All data generated in this research were reported in the manuscript.
